# Supplementary material for: The microbiota of moon snail egg collars is shaped by host-specific factors
Source: Microbiol Spectr. 2024 Oct 4;12(11):e01804-24. doi: 10.1128/spectrum.01804-24 (PMC11537117; doi:10.1128/spectrum.01804-24)
Supplement: Supplemental figures and tables — Tables S1 to S11; Fig. S1 to S8. [file spectrum.01804-24-s0001.docx]

Supplemental Information

**The Microbiota of Moon Snail Egg Collars is Shaped by Host-Specific Factors**

*Karla Piedl,^a^ Frank O. Aylward,^b^ Emily Mevers^a,^#*

^a^Department of Chemistry, Virginia Tech, Blacksburg, Virginia, USA

^b^Department of Biological Sciences, Virginia Tech, Blacksburg, Virginia, USA

**Table of Contents**

**Figure S1:** Location of collection sites in Pine Island Sound Aquatic Reserve, Florida ………………….3

**Figure S2:** Stacked bar plots of relative abundance for phylum, class, order and family taxonomy (February 2023) ………………………………………………………………………………………………………..4

**Figure S3:** NMDS plots for egg collar phylum, class, order and family taxonomy (February 2023) ……..5

**Figure S4:** NMDS plots for sediment phylum and genus taxonomy (February 2023) ................................6

**Table S1:** F values and R squared values for PERMANOVA analysis…………………………………….7

**Table S2:** metadata collected from 2023 field collection…………………………………………………..8

**Figure S5:** Top 20 stacked bar plots, December/January/February; NMDS plot of collar collections by month…………………….…………………………………………………………………………………9

**Table S3:** F values and R squared values for 2024 collection PERMANOVA analysis………………….10

**Table S4:** OTU total counts for fragile vs. firm collars…………………………………………………...11

**Table S5:** The variable core (bacteria present on 50-80%) of egg collars collected in 2023.……….……12

**Table S6:** Core microbiota of sediment samples………………………………………………………….14

**Table S7:** Core microbiota of samples collected in Dec/Jan/Feb…………………………………………15

**Table S8:** Core microbiota of fragile egg collars samples………………………………………………...16

**Table S9:** Core microbiota of firm egg collars samples…………………………………………………..17

**Figure S6:** Species richness at phylum, class, order and family levels (bar plots) ……………………….19

**Table S10:** Hill values table………………………………………………………………………………20

**Figure S7:** DESeq2 volcano plot………………………………………………………………………….21

**Table S11:** MIBiG matches to most abundant AD sequences from the egg collars………….…………..22

**Figure S8**: Partial 18S rRNA alignment between sequences from *Neverita delessertiana*, *N. duplicata*, and 2023 collected egg collars……………………………………………………………………………….....23


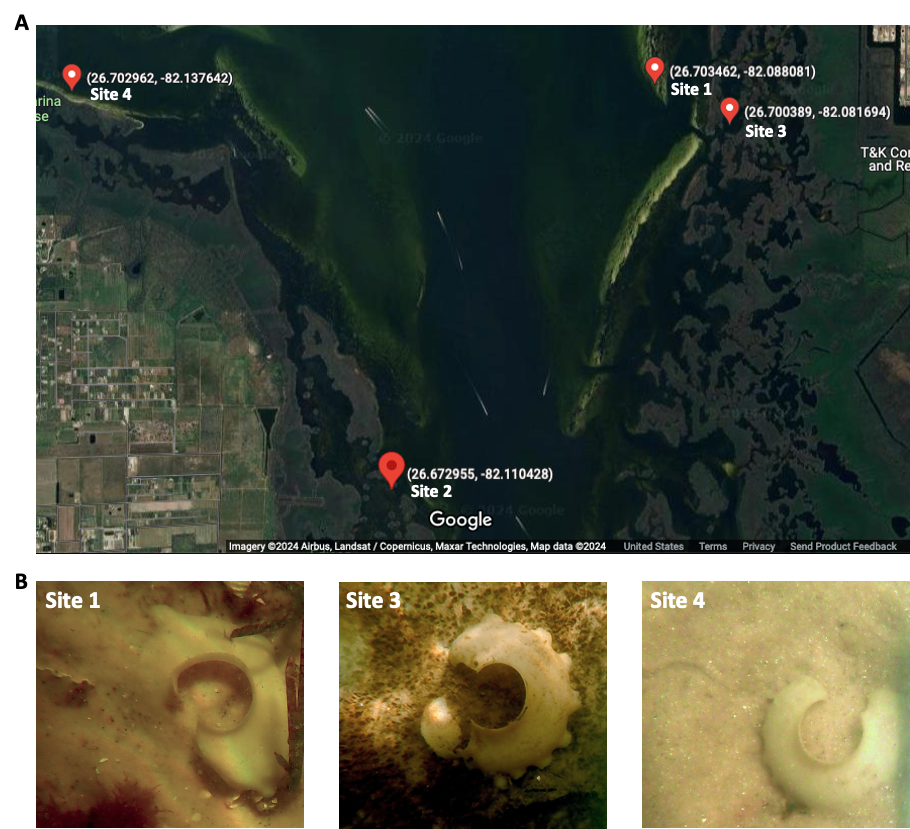


**Figure S1** - Location of collection sites in Pine Island Sound Aquatic Reserve, SW Florida, with underwater pictures of egg collars taken at sites 1, 3 and 4 showcasing the different sediment types. Examples are shown of the different sand found at the different sites. Sand bars, like site 1, are indicated by the light green color. Sites 2 and 3 had similar sand types and were dominated by mangrove trees with a swampy sand texture. Site 4 was a sandy beach at the north end of Pine Island.


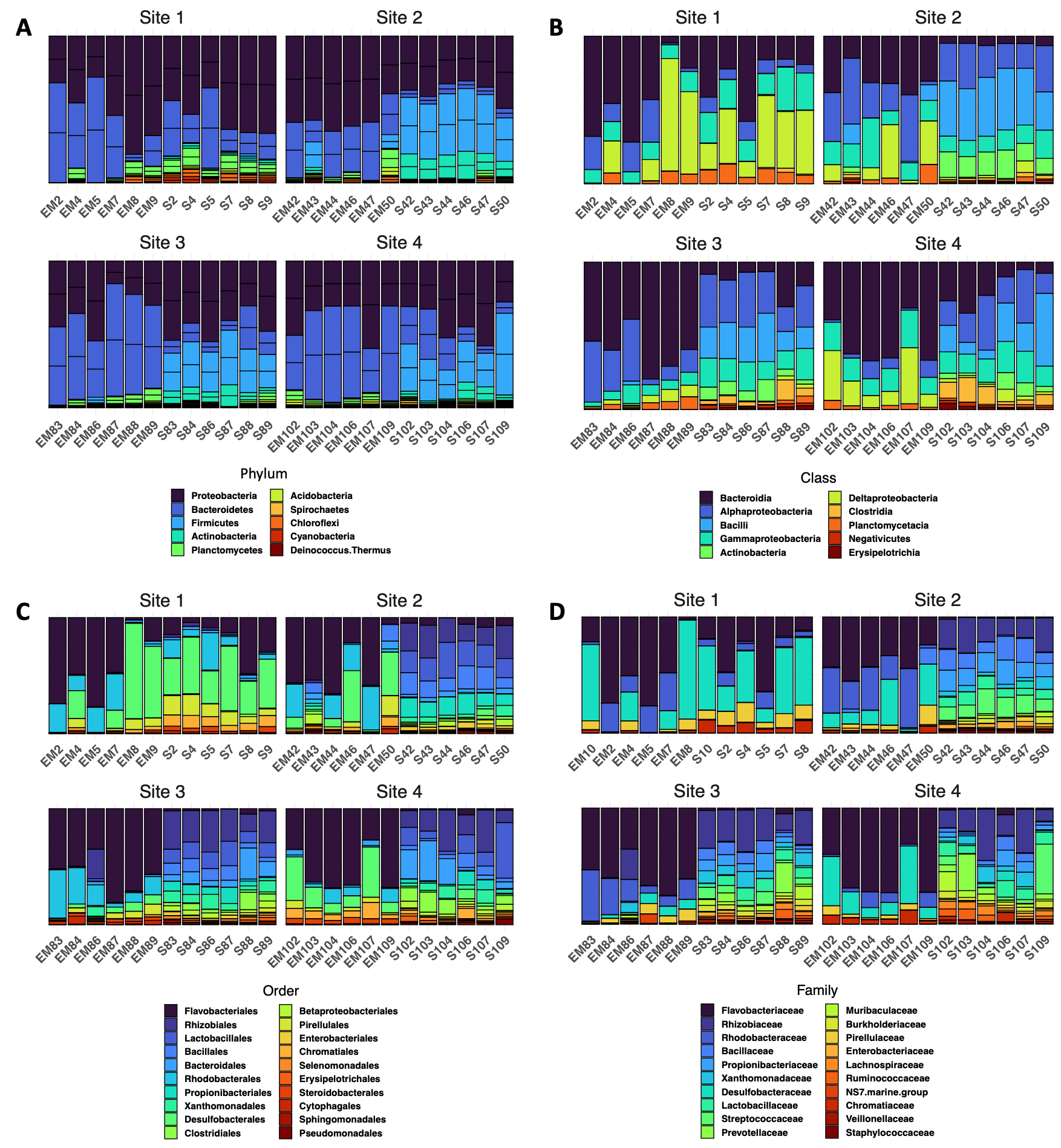


**Figure S2** - Relative abundance stacked bar plots of top 10 most abundant taxonomy units in egg collar and sediment samples at (**A**) Phylum, (**B**) Class levels, and top 20 most abundant taxonomy units for egg collar and sediment samples at (**C**) Order, and (**D**) Family levels.


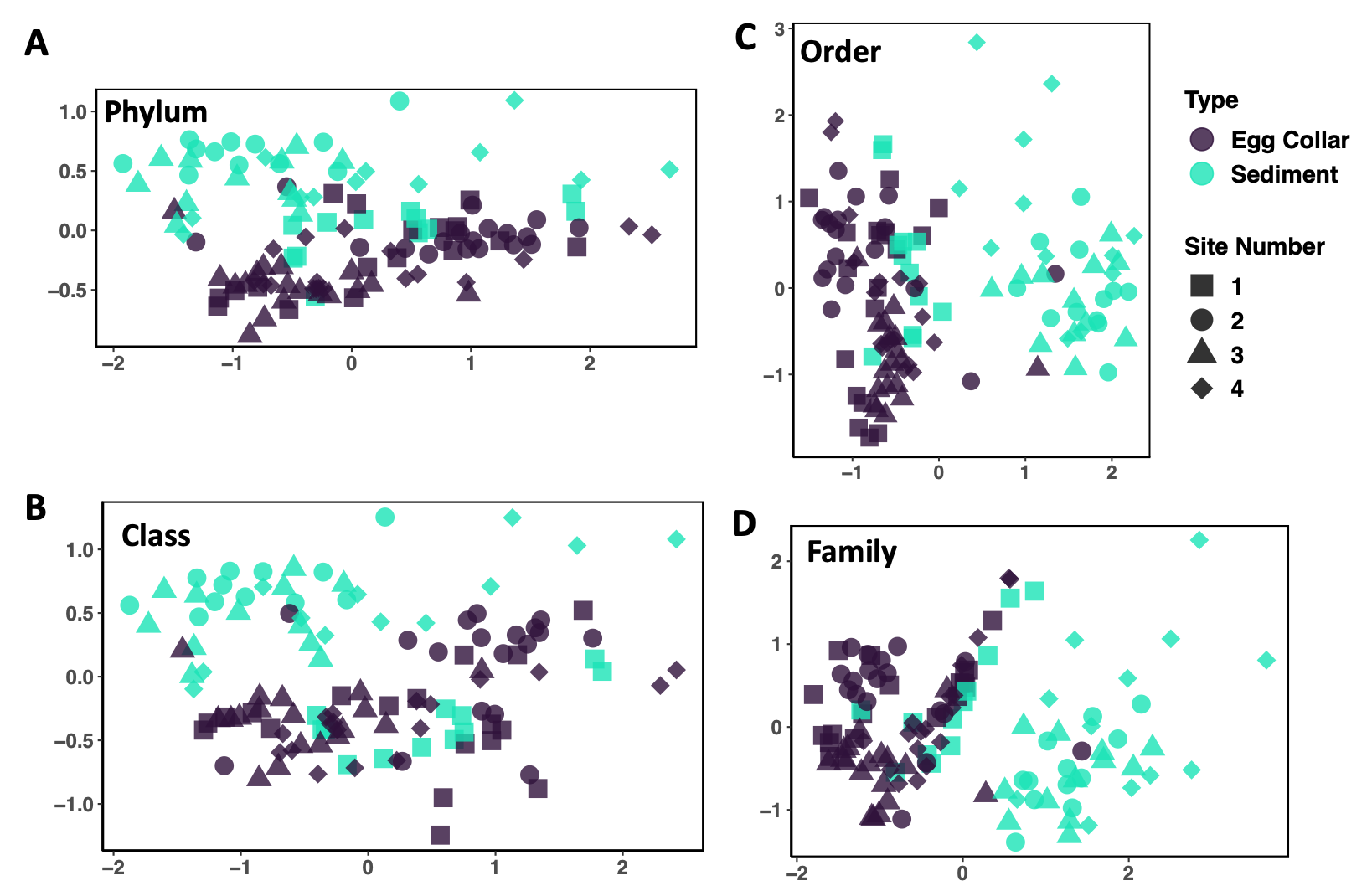


**Figure S3** -NMDS plots at Phylum (**A**), Class (**B**), Order (**C**), and Family (**D**) taxonomic levels. For all plots, egg collars are shown in dark purple; sediment samples are shown in teal. Different site numbers are indicated by shape. Distinct clustering is seen at all taxonomic levels (p-values are 0.005, <0.001, <0.001, and <0.001 for phylum, class, order, and family levels respectively).


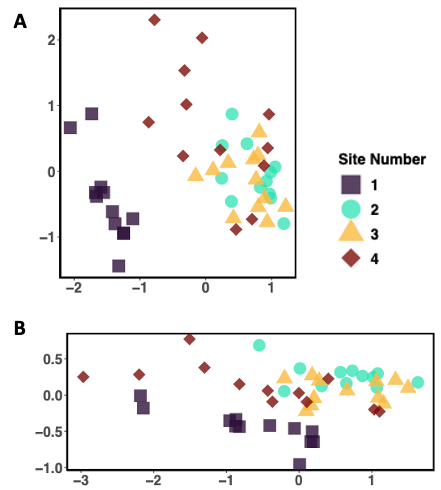


**Figure S4** - NMDS plots at (**A**) Phylum and (**B**) Genus taxonomy levels for sediment samples clustered by site number. Each color/shape represents a different site. Clustering by site number and sediment type is seen at both levels - site 1 (sand bar) clusters alone, sites 2 and 3 (mangrove bay, mucky bottom) cluster together, and site 4 (sandy beach) clusters with itself (p-values are <0.001 for both phylum and genus levels).

| **Table S1** - F values, R squared values for PERMANOVA analysis, 2023 collection trip | | | | |
| --- | --- | --- | --- | --- |
| **Egg Collars v. Sand** | | | | |
|  | **Sum of Squares** | **R squared** | **F** | **P-Value** |
| Phylum | 1.386514 | 0.139654 | 7.466872 | >0.001 |
| Class | 1.429263 | 0.125158 | 6.580938 | >0.001 |
| Order | 2.835513 | 0.195217 | 11.158227 | >0.001 |
| Family | 2.859572 | 0.190965 | 10.857875 | >0.001 |
| Genus | 2.822268 | 0.180223 | 10.112825 | >0.001 |
| **Egg Collars vs. Texture^1^** | | | | |
|  | **Sum of Squares** | **R squared** | **F** | **P-Value** |
| Phylum | 0.5488 | 0.044881 | 1.0634 | 0.332 |
| Class | 0.8133 | 0.0558 | 1.3395 | 0.196 |
| Order | 0.8963 | 0.5386 | 1.2903 | 0.214 |
| Family | 0.975 | 0.05706 | 1.3717 | 0.161 |
| Genus | 2.1644 | 0.35632 | 3.69051 | >0.001 |
| **Egg Collars vs. Site^1^** | | | | |
|  | **Sum of Squares** | **R squared** | **F** | **P-Value** |
| Phylum | 0.996589 | 0.2440994 | 2.152835 | 0.079 |
| Class |  |  |  |  |
| Order | 1.442262 | 0.259962 | 2.341886 | 0.019 |
| Family |  |  |  |  |
| Genus | 1.661937 | 0.273581 | 2.510769 | 0.007 |
| **Sediment vs. Site^2^** | | | | |
|  | **Sum of Squares** | **R squared** | **F** | **P-Value** |
| Phylum | 1.7608 | 0.39487 | 4.3503 | >0.001 |
| Class | 2.3102 | 0.45022 | 5.4595 | >0.001 |
| Order | 3.1187 | 0.50784 | 6.8789 | >0.001 |
| Family | 3.2095 | 0.5000 | 6.6666 | >0.001 |
| Genus | 3.2911 | 0.48667 | 6.3204 | >0.001 |

^1^ Only egg collar samples were used for these calculations; ^2^ Only sediment samples were used for these calculations

| **Table S2** - Collected data for sequenced egg masses. Samples are separated by site; egg collar texture is highlighted in bold. | | | | | | | | | | | |
| --- | --- | --- | --- | --- | --- | --- | --- | --- | --- | --- | --- |
| **Name** | **Site Num^1^** | **Water Temp** | **Date** | **Depth (cm)** | **Size (cm)** | **Crabs** | **Snail** | **Algae** | **Biofilm** | **Texture** | **Color** |
| **EM2** | 1 | 74.6 | 2/2/23 | 50 | 10 | 0 | 1 | 0 | 0 | **4** | 3 |
| **EM4** | 1 | 74.6 | 2/2/23 | 58 | 9 | 0 | 1 | 0 | 0 | **2** | 1 |
| **EM5** | 1 | 74.6 | 2/2/23 | 65 | 9 | 0 | 1 | 0 | 0 | **4** | 3 |
| **EM7** | 1 | 74.6 | 2/2/23 | 65 | 7 | 0 | 1 | 0 | 0 | **1** | 1 |
| **EM8** | 1 | 74.6 | 2/2/23 | 50 | 10 | 1 | 0 | 0 | 0 | **1** | 1 |
| **EM9** | 1 | 74.6 | 2/2/23 | 80 | 9 | 0 | 0 | 0 | 0 | **1** | 1 |
|  | | | | | | | | | | | |
| **EM42** | 2 | 74.6 | 2/2/23 | 65 | 9 | 0 | 1 | 0 | 0 | **2** | 2 |
| **EM43** | 2 | 74.6 | 2/2/23 | 65 | 13 | 0 | 1 | 0 | 0 | **2** | 2 |
| **EM44** | 2 | 74.6 | 2/2/23 | 65 | 9 | 0 | 1 | 0 | 0 | **2** | 2 |
| **EM46** | 2 | 74.6 | 2/2/23 | 80 | 7.5 | 0 | 1 | 0 | 0 | **2** | 2 |
| **EM47** | 2 | 74.6 | 2/2/23 | 65 | 10.5 | 0 | 1 | 0 | 0 | **2** | 2 |
| **EM50** | 2 | 74.6 | 2/2/23 | 65 | 12.5 | 0 | 1 | 1 | 1 | **3** | 3 |
|  | | | | | | | | | | | |
| **EM83** | 3 | 74.9 | 2/3/24 | 58 | 6.5 | 0 | 1 | 0 | 1 | **4** | 3 |
| **EM84** | 3 | 74.9 | 2/3/24 | 50 | 7 | 0 | 1 | 0 | 0 | **3** | 2 |
| **EM86** | 3 | 74.9 | 2/3/24 | 65 | 6.5 | 0 | 1 | 0 | 0 | **4** | 3 |
| **EM87** | 3 | 74.9 | 2/3/24 | 58 | 5.5 | 0 | 1 | 0 | 0 | **3** | 3 |
| **EM88** | 3 | 74.9 | 2/3/24 | 55 | 9 | 0 | 1 | 0 | 1 | **2** | 2 |
| **EM89** | 3 | 74.9 | 2/3/24 | 55 | 9 | 1 | 1 | 0 | 0 | **2** | 2 |
|  | | | | | | | | | | | |
| **EM102** | 4 | 74.9 | 2/3/24 | 50 | 8.5 | 0 | 0 | 0 | 0 | **1** | 1 |
| **EM103** | 4 | 74.9 | 2/3/24 | 65 | 9.5 | 0 | 0 | 0 | 0 | **1** | 1 |
| **EM104** | 4 | 74.9 | 2/3/24 | 40 | 6 | 0 | 0 | 0 | 0 | **1** | 1 |
| **EM106** | 4 | 74.9 | 2/3/24 | 55 | 7 | 0 | 0 | 0 | 0 | **1** | 1 |
| **EM107** | 4 | 74.9 | 2/3/24 | 65 | 7 | 0 | 0 | 0 | 0 | **1** | 1 |
| **EM109** | 4 | 74.9 | 2/3/24 | 55 | 9 | 0 | 0 | 0 | 0 | **1** | 1 |

^1^Site 1 is a sandbar (82°-5’ 17.09” W 26°-42’ 12.462” N), site 2 and 3 are mangrove bays (82°-6’ 37.54” W 26°-40’ 22.638” N and 82°-4’ 54.1” W 26°-42’ 1.4” N, respectively), and site 4 is a sandy bottom beach (82°-8’ 15.51” W 26°-42’ 10.662” N).


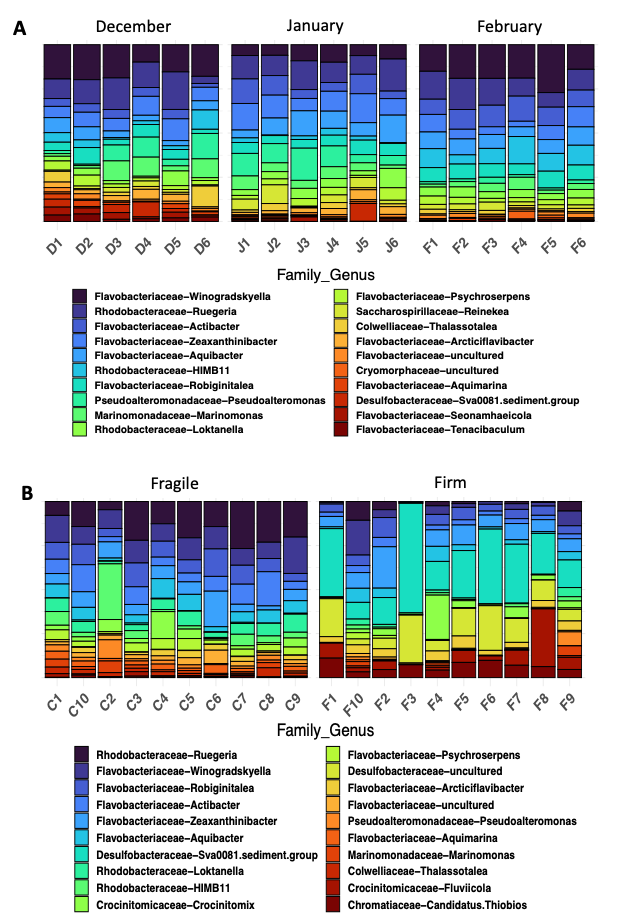


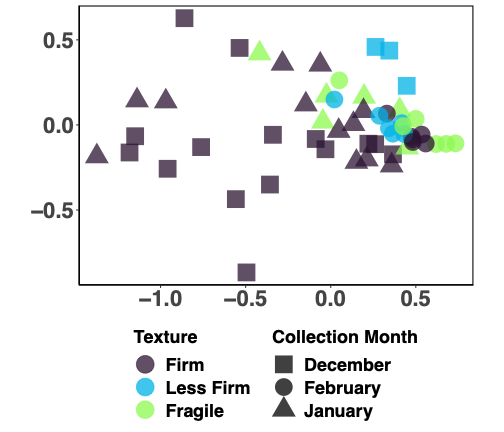


**Figure S5** - (**A**) Relative abundance of bacterial genera from egg collars collected in December (D), January (J) and February (F). Each collar was sequenced in triplicate. The relative abundance was calculated from the average of the sequencing runs. Different colors indicate different genera, height of bars indicate relative abundance of genera in that sample. (**B**) NMDS plot of Bray-Curtis diversity distances for collars collected between December and February.

| **Table S3** - F values, R squared values for PERMANOVA analysis. All analysis performed at the genus level. | | | | |
| --- | --- | --- | --- | --- |
| **December, January, February** | | | | |
|  | **Sum of Squares** | **R squared** | **F** | **P-Value** |
| **By Month** | 1.9705 | 0.26613 | 9.2473 | <0.0001 |
| **By Texture** | 0.8708 | 0.11761 | 3.3988 | 0.005 |
| **Firm vs. Fragile** | | | | |
|  | **Sum of Squares** | **R squared** | **F** | **P-Value** |
| **By Texture** | 7.7160 | 0.5613 | 74.208 | < 0.001 |

| **Table S4 -** Total abundance for top ten genera present on egg collars samples. All genera listed are members of the core80 microbiome. | |
| --- | --- |
| **Egg Collars** | |
| **Genus** | **Abundance Count** |
| *Zeaxanthinibacter* | 390,943 |
| Sva0081 Sediment Group | 88,525 |
| *Ruegeria* | 80,699 |
| *Winogradskyella* | 79,450 |
| Uncultured NS7 Marine Group bacteria | 77,701 |
| Uncultured Desulfobacteraceae | 76,116 |
| *Robiginitalea* | 68,179 |
| *Actibacter* | 56,970 |
| *Arcticflavibacter* | 36,141 |
| Uncultured Flavobacteriaceae | 35,836 |

| **Table S5 -** The variable core (bacteria present on 50-80%) of egg collars collected in 2023. | | | | | |
| --- | --- | --- | --- | --- | --- |
| **Phylum** | **Class** | **Order** | **Family** | **Genius** | **Count** |
| Actinobacteria | Acidimicrobiia | Actinomarinales | Uncultured^2^ | Uncultured^2^ | 18 |
|  |  | Microtrichales | Ilumatobacteraceae | *Ilumatobacter* | 32 |
| Bacteroidota (Bacteriodetes)^1^ | Bacteroidia | Bacteroidales | Marinilabiliaceae | Uncultured^2^ | 34 |
|  |  | Bacteroidetes  VC2.1 Bac22 | Uncultured^2^ |  | 25 |
|  |  | Chitinophagales | Saprospiraceae | Uncultured^2^ | 29 |
|  | Flavobacteriia | Flavobacteriales | Crocinitomicaceae | *Crocinitomix* | 17 |
|  |  |  | Cryomorphaceae | Uncultured^2^ | 17 |
|  |  |  | Flavobacteriaceae | *Dokdonia* | 27 |
|  | Ignavibacteria | Ignavibacteriales | Ignavibacteriaceae | *Ignavibacterium* | 33 |
|  |  |  | Melioribacteraceae | *IheB3-7* | 34 |
|  | Calditrichia | Calditrichales | Calditrichaceae | *Caldithrix* | 30 |
|  |  |  |  | *Calorithrix* | 30 |
| Cyanobacteria | Oxyphoto-  bacteria | Nostocales | Cyanobacteriaceae | *Cyanobacterium CLg1* | 29 |
|  |  | Synechococcales | Cyanobiaceae | *Cyanobium PCC 6307* | 27 |
| Desulfobacterota^4^ | Desulfobacteria^1^ | Desulfobacterales | Desulfobacteraceae | *Desulfococcus* | 24 |
|  |  |  |  | SEEP.SRB1 | 19 |
| Gemmatimonadota (Gemmatimonadetes)^1^ | PAUC43f Marine Benthic Group^1^ | Uncultured^2^ |  |  | 30 |
| Latescibacterota (Latescibacteria)^4^ | Uncultured^2^ |  |  |  | 20 |
|  | Uncultured^2^ |  |  |  | 34 |
| Myxococcota^1^ | Myxococcia | Myxococcales | Sandaracinaceae | Uncultured^2^ | 26 |
| Planctomycetota (Planctomycetes)^1^ | OM190 | Uncultured^2^ |  |  | 34 |
|  | Planctomyce-  tacia | Pirellulales | Pirellulaceae | Pir4 lineage | 23 |
|  |  |  |  | *Rubripirellula* | 22 |
|  |  |  |  | Uncultured^2^ | 29 |
| Pseudomonadota (Proteobacteria)^1^ | Alphaproteo-  bacteria | Rhizobiales | Rhizobiaceae | *Lentilitoribacter* | 26 |
|  |  |  |  | *Ochrobactrum* | 32 |
|  |  | Rhodobacterales | Rhodobacteraceae | *Loktanella* | 20 |
|  |  |  |  | *Nautella* | 32 |
|  |  |  |  | *Roseivivax* | 29 |
|  |  |  |  | *Thalassobius* | 34 |
|  |  |  |  | Uncultured^2^ | 31 |
|  |  | Rhodospirillales | Rhodopirillaceae | *Defluviicoccus* | 31 |
|  | Gammaproteo-  bacteria | Alteromonadales | Colwelliaceae | *Thalassotalea* | 31 |
|  |  | Arenicellales | Arenicellaceae | Uncultured^2^ | 27 |
|  |  | Cellvibrionales | Halieaceae | *Halioglobus* | 18 |
|  |  |  |  | *Pseudohaliea* | 34 |
|  |  | Chromatiales | Sedimenticolaceae | *Sedimenticola* | 32 |
|  |  | Ectothiorhodospirales | Ectothiorhodospiraceae | *Thiogranum* | 20 |
|  |  | HOC36 | Uncultured^2^ |  | 29 |
|  |  | KI89A.clade | Uncultured^2^ |  | 30 |
|  |  | Nitrosococcales | Nitrosococcaceae | CI75cm.2.12 | 20 |
|  |  | Oceanospirillales | Pseudohongiellaceae | *Pseudohongiella* | 28 |
|  |  | Thiotrichales | Thiotrichaceae | Uncultured^2^ | 29 |
| Spirochaetes | Spirochaetia | Spirochaetales | Spirochaetaceae | *Sediminispirochaeta* | 34 |
|  |  |  |  | Spirochaeta 2 | 21 |

^1^Taxonomy reflects the current accepted bacterial nomenclature; previously used names are indicated in parenthesis [(Waite et al. 2020; Langwig et al. 2022; Oren and Garrity 2021; Aldeguer-Riquelme et al, 2023 )](https://paperpile.com/c/uTGU2r/nzzj+eHGc+hXtu); ^2^Uncultured indicates the organism has not been cultured in a laboratory setting and that taxonomy has not been assigned (as of Silva v. 138 - 2019); ^3^Candidatus indicates the genus has been described but no species have been cultured in the lab; ^4^Phylum still has candidatus status [(Gavriilidou et al. 2023)](https://paperpile.com/c/uTGU2r/Yic8). ^5^

| **Table S6** - Core microbiota of sediment samples | | | | | |
| --- | --- | --- | --- | --- | --- |
| **Core80 (genera present on ≥ 80% samples)** | | | | | |
| **Phylum^1^** | **Class** | **Order** | **Family** | **Genus** | **Count** |
| Actino-  bacteria | Actino-  bacteria | Propioni-  bacteriales | Propioni-  bacteriaceae | *Cutibacterium* | 24 |
| Bacteroidota  (Bacteroidetes) | Bacteroidia | Bacteroidales | Muribaculaceae | Uncultured^2^ | 21 |
|  | Flavobacteriia | Flavobacteriales | NS7 Marine Group | Uncultured^2^ | 22 |
| Firmicutes | Bacilli | Bacillales | Bacillaceae | *Bacillus* | 21 |
|  |  |  | Staphylococcaceae | *Staphylococcus* | 20 |
|  |  | Lactobacillales | Lactobacillaceae | *Lactobacillus* | 22 |
| Pseudomonadota (Proteobacteria) | Alpha-  proteobacteria | Rhizobiales | Rhizobiaceae | *Ochrobactrum* | 24 |
|  | Gamma-  proteobacteria | Xantho-  monadales | Xanthomona-  daceae | *Pseudoxantho-*  *monas* | 22 |
| **Variable core (genera present on 50-80% of samples)** | | | | | |
| **Phylum^1^** | **Class** | **Order** | **Family** | **Genus** | **Count** |
| Actinobacteria | Actinobacteria | Propionibacteriales | Propionibacteriaceae | *Cutibacterium* | 7 |
| Bacteroidetes | Bacteroidia | Bacteroidales | Muribaculaceae | Uncultured | 9 |
|  | Flavobacteriia | Flavobacteriales | Flavobacteriaceae | *Actibacter* | 11 |
|  |  |  |  | *Zeaxanthinibacter* | 11 |
| Desulfobacterota^4^ | Desulfobacteria^1^ | Desulfobacterales | Desulfobacteraceae | Sva0081 | 7 |
|  |  |  |  | Uncultured | 10 |
| Firmicutes | Bacilli | Bacillales | Bacillaceae | *Bacillus* | 8 |
|  |  |  | Staphylococcaceae | *Staphylococcus* | 7 |
|  |  | Lactobacillales | Lactobacillaceae | *Lactobacillus* | 8 |
|  |  |  | Streptococcaceae | *Lactococcus* | 9 |
|  |  |  |  | *Streptococcus* | 10 |
|  | Erysipelotrichia | Erysipelotrichales | Erysipelotrichaceae | *Turicibacter* | 9 |
| Proteobacteria | Alphaproteo-  bacteria | Sphingomonadalea | Sphingomonadaceae | *Novosphingobium* | 10 |
|  | Gammaproteo-  bacteria | Chromatiales | Chromatiaceae | *Ca.* Thiobios | 9 |
|  |  | Enterobacteriales | Enterobacteriaceae | *Escherichia/Shigella* | 9 |
|  |  | Xanthomonadales | Xanthomonadaceae | *Pseudoxanthomonas* | 8 |
| Spirochaetes | Leptospirae | Leptospirales | Leptospiraceae | *Turneriella* | 9 |

^1^Taxonomy reflects the current accepted bacterial nomenclature; previously used names are indicated in parenthesis [(36–38)](https://paperpile.com/c/uTGU2r/nzzj+eHGc+hXtu); ^2^Uncultured indicates the organism has not been cultured in a laboratory setting and that taxonomy has not been assigned (as of Silva v. 138 - 2019); ^3^Candidatus indicates the genus has been described but no species have been cultured in the lab; ^4^Phylum still has candidatus status [(39)](https://paperpile.com/c/uTGU2r/Yic8); ^5^Originally labeled *incertae sedis* (latin for ‘of uncertain placement’)

| **Table S7** - Core microbiota of moon snail egg collars collected in December 2023 and January and February 2024 | | | | | |
| --- | --- | --- | --- | --- | --- |
| **Core80 (genera present on ≥ 80% samples)** | | | | | |
| **Phylum** | **Class** | **Order** | **Family** | **Genus** | **Count** |
| Bacteroidota (Bacteriodetes) | Flavobacteriia | Flavobacteriales | Flavobacteriaceae | *Actibacter* | 0 |
|  |  |  |  | *Aquibacter* | 0 |
|  |  |  |  | *Arcticflavibacter* | 1 |
|  |  |  |  | *Psychroserpens* | 2 |
|  |  |  |  | *Robiginitalea* | 0 |
|  |  |  |  | *Winogradskyella* | 0 |
|  |  |  |  | *Zeaxanthinibacter* | 0 |
| Pseudomonadota (Proteobacteria) | Alphaproteo-  bacteria | Rhodobacterales | Rhodobacteraceae | *Loktanella* | 1 |
|  |  |  |  | *Ruegeria* | 0 |
|  | Gammaproteo-  bacteria | Alteromonadales | Pseudoalteromon-  adaceae | *Pseudoalteromonas* | 0 |
|  |  | Oceanospirillales | Marinomonadaceae | *Marinomonas* | 0 |
|  |  |  | Saccharospirillaceae | *Reinekea* | 1 |
| **Variable core (genera present on 50-80% of samples)** | | | | | |
| **Phylum** | **Class** | **Order** | **Family** | **Genus** | **Count** |
| Bacteroidota  (Bacteriodetes) | Cytophagia | Cytophagales | Cyclobacteriaceae | Uncultured | 13 |
|  | Flavobacteriia | Flavobacteriales | Cryomorphaceae | Uncultured | 9 |
|  |  |  | Flavobacteriaceae | *Aquimarina* | 12 |
|  |  |  |  | Uncultured | 12 |
| Pseudomonadota  (Proteobacteria) | Alphaproteo-  bacteria | Rhodobacterales | Rhodobacteraceae | HIMB11 | 13 |
|  | Gammaproteo-  bacteria | Alteromonadales | Colwelliaceae | *Thalassotela* | 14 |
|  |  |  | Pseudoalteromon-  adaceae | *Psychrosphaera* | 9 |

| **Table S8** - Core microbiota of fragile moon snail egg collars; genera present on ≥ 80% of egg collars | | | | | |
| --- | --- | --- | --- | --- | --- |
| **Core80 (genera present on ≥ 80% samples)** | | | | | |
| **Phylum** | **Class** | **Order** | **Family** | **Genus** | **Count** |
| Bacteroidota (Bacteriodetes) | Flavobacteriia | Flavobacteriales | Crocinitomicaceae | *Crocinitomix* | 2 |
|  |  |  | Flavobacteriaceae | *Actibacter* | 10 |
|  |  |  |  | *Aquibacter* | 10 |
|  |  |  |  | *Aquimarina* | 9 |
|  |  |  |  | *Psychroserpens* | 9 |
|  |  |  |  | *Robiginitalea* | 10 |
|  |  |  |  | *Winogradskyella* | 10 |
|  |  |  |  | *Zeaxanthinibacter* | 10 |
|  |  |  |  | Uncultured | 10 |
| Pseudomonadota (Proteobacteria) | Alphaproteo-  bacteria | Rhodobacterales | Rhodobacteraceae | HIMB11 | 8 |
|  |  |  |  | *Loktanella* | 9 |
|  |  |  |  | *Ruegeria* | 10 |
|  | Gammaproteo-  bacteria | Alteromonadales | Colwelliaceae | *Thalassotalea* | 8 |
| **Variable core (genera present on 50-80% of samples)** | | | | | |
| **Phylum** | **Class** | **Order** | **Family** | **Genus** | **Count** |
| Bacteroidota (Bacteriodetes) | Flavobacteriia | Flavobacteriales | Flavobacteriaceae | *Arctiflavibacter* | **7** |
| Pseudomonadota  (Proteobacteria) | Alphaproteo-  bacteria | Rhodobacterales | Rhodobacteraceae | *Ascidiaceihabitans* | 6 |
|  |  |  |  | *Nautella* | 5 |
|  |  |  | Roseobacteraceae | *Roseovarius* | 6 |
|  | Gammaproteo-  bacteria | Alteromonadales | Pseudoalteromon-  adaceae | *Pseudoalteromonas* | 7 |
|  |  | Oceanospirillales | Oceanospirillaceae | *Marinomonas* | 6 |

| **Table S9** - Core microbiota of firm moon snail egg collars; genera present on ≥ 80% of egg collars | | | | | |
| --- | --- | --- | --- | --- | --- |
| **Core80 (genera present on ≥ 80% samples)** | | | | | |
| **Phylum** | **Class** | **Order** | **Family** | **Genus** | **Count** |
| Acidobacteriota (Acidobacteria) | Thermoanaero-  baculia | Thermoanaero-  baculales | Thermoanaero-  baculaceae | Subgroup 23 | 9 |
| Bacteroidota (Bacteriodetes) | Bacteroidia | Bacteroidales | Bacteroidetes BD2-2 | Uncultured | 8 |
|  | Cytophagia | Cytophagales | Cyclobacteriaceae | Uncultured | 10 |
|  | Flavobacteriia | Flavobacteriales | Crocinitomicaceae | *Fluviicola* | 9 |
|  |  |  | Flavobacteriaceae | *Actibacter* | 9 |
|  |  |  |  | *Arcticiflavibacter* | 9 |
|  |  |  |  | *Aquibacter* | 8 |
|  |  |  |  | *Robiginitalea* | 9 |
|  |  |  |  | *Winogradskyella* | 9 |
|  |  |  |  | *Zeaxanthinibacter* | 9 |
| Chloroflexota  (Chloroflexi) | Anaerolineae | Anaerolineales | Anaerolineaceae | Uncultured | 10 |
| Desulfobacterota^4^ | Desulfobacteria | Desulfobacterales | Desulfobacteraceae | Sva0081 | 10 |
|  |  |  |  | Uncultured^2^ | 10 |
|  |  |  |  | *Desulfosarcina* | 9 |
| Planctomyetota  (Planctomycetes) | Plancomycetia | Pirellulales | Pirellulaceae | *Pirellula* | 9 |
| Pseudomonadota (Proteobacteria) | Gammaproteo-  bacteria | Chromatiales | Chromatiaceae | *Candidatus* Thiobus^3^ | 10 |
| Thermodesulfo-  bacteriota | Syntropho-  bacteria | Syntropho-  bacterales | Syntropho-  bacteraceae | Uncultured | 8 |
| **Variable core (genera present on 50-80% of samples)** | | | | | |
| **Phylum** | **Class** | **Order** | **Family** | **Genus** | **Count** |
| Bacteriodota  (Bacteriodota) | Flavobacteriia | Flavobacteriales | Crocinitomicaceae | *Crocinitomix* | 7 |
|  |  |  | Flavobacteriaceae | *Aquibacter* | 7 |
|  |  |  |  | *Wenyingzhuangia* | 6 |
| Calditrichota | Calditrichia | Calditrichales | Calditrichaceae | Uncultured | 7 |
| Desulfobacterota | Desulfobacteria | Deasulfobacterales | Desulfosarcinaceae | SEEP SRB1 | 7 |
| Planctomycetota | Plactomycetia | Pirellulales | Pirellulaceae | Pir 4 lineage | 7 |
|  |  |  |  | *Rhodopirellula* | 7 |
| Pseudomonadota  (Proteobacteria) | Gammaproteo-  bacteria | Chromatiales | Woeseiaceae | *Woeseia* | 5 |
| Spirochaetota | Spirochaetia | Spirochaetales | Spirochaetaceae | Spirochaeta2 | 6 |

^1^Taxonomy reflects the current accepted bacterial nomenclature; previously used names are indicated in parenthesis (Waite et al. 2020; Langwig et al. 2022; Oren and Garrity 2021); ^2^Uncultured indicates the organism has not been cultured in a laboratory setting and that taxonomy has not been assigned (as of Silva v. 138 - 2019); ^3^Candidatus indicates the genus has been described but no species have been cultured in the lab


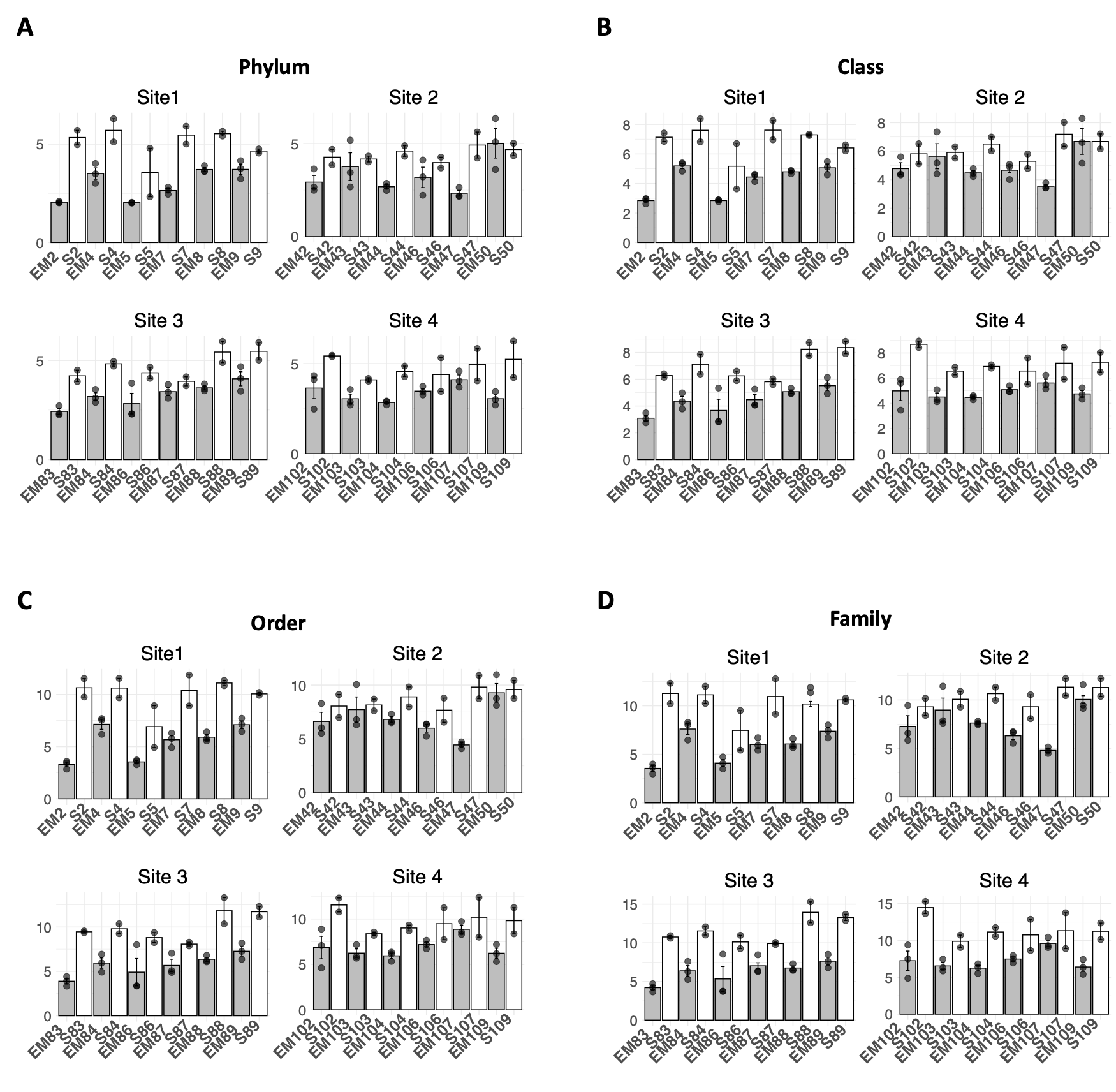


**Figure S6** -Bar plots of species richness for all taxonomic levels - Phylum (**A**), Class (**B**), Order (**C**), and Family (**D**)

| **Table S10** Hill values and site averages for samples separated by sample type (egg mass vs. sand) | | | | | |
| --- | --- | --- | --- | --- | --- |
| **Site 1** | | | **Site 2** | | |
|  | **Species Richness** | |  | **Species Richness** | |
| **Sample** | **Egg Mass** | **Sand** | **Sample** | **Egg Mass** | **Sand** |
| 2 | 32.03554^#^ | 107.69773 | 42 | 53.08420^Ꞩ^ | 74.39798 |
| 4 | 68.48800^Ꞩ^ | 112.49479 | 43 | 64.46797^Ꞩ^ | 69.28575 |
| 5 | 36.76581^#^ | 74.65616 | 44 | 53.80826^Ꞩ^ | 86.53386 |
| 7 | 50.00200 | 95.64740 | 46 | 55.36696^Ꞩ^ | 63.07553 |
| 8 | 61.35404 | 95.66206 | 47 | 46.02906^Ꞩ^ | 94.01904 |
| 9 | 65.55247 | 96.97357 | 50 | 91.49746^‡^ | 78.79662 |
| **Average** | 52.36631 | 97.18862 | **Average** | 60.70899 | 77.68480 |
| **Site 3** | | | **Site4** | | |
|  | **Species Richness** | |  | **Species Richness** | |
| **Sample** | **Egg Mass** | **Sand** | **Sample** | **Egg Mass** | **Sand** |
| 83 | 50.67416^#^ | 61.34544 | 102 | 47.80586 | 111.99605 |
| 84 | 69.57901^‡^ | 86.47954 | 103 | 54.32494 | 77.50280 |
| 86 | 50.33261^#^ | 74.64003 | 104 | 64.34844 | 71.59930 |
| 87 | 68.27803^‡^ | 46.83252 | 106 | 77.97223 | 68.54670 |
| 88 | 75.65465 | 132.91503 | 107 | 92.03302 | 90.18468 |
| 89 | 72.07601 | 116.56359 | 109 | 69.75941 | 56.38298 |
| **Average** | 64.43241 | 86.46269 | **Average** | 67.70732 | 79.36875 |

^Ꞩ^ Egg collars have a mostly firm texture; ^‡^ Egg collars have a mostly fragile texture; ^#^ Egg collars have a very fragile texture.


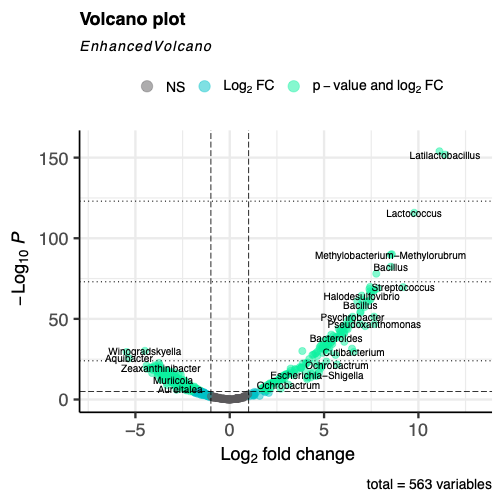


**Figure S7** - ASV count taxonomic enrichment of egg mass vs. sand samples. Grey dots indicate non-statistically significant genera, blue dots indicate non-significant fold changes, green dots indicate statistically significant fold changes. P-value = 10e-6, fold change = 0.5.

| **Table S11** - MIBiG matches to most abundant AD sequences from the egg collars, currently organized by abundance^1, 2^ | | | | | | |
| --- | --- | --- | --- | --- | --- | --- |
|  | **Identity (%)** | **E-value** | **Compound** | **Compound Type** | **Gene Match** | **Function** |
| BGC0001343 | 29.5 | 2.81E-06 | aurachin A | PKS | auaEII^3^ | antibiotic |
| BGC0001067 | 43.2 | 4.25E-21 | fumagillin | PKS  Terpene | AFUA_8G00500^3^ | antifungal, amebicide |
|  | 46.3 | 2.14E-23 |  |  |  |  |
|  | 46.6 | 3.12E-17 |  |  |  |  |
|  | 46.6 | 3.12E-17 |  |  |  |  |
|  | 46.6 | 3.12E-17 |  |  |  |  |
|  | 46.6 | 3.12E-17 |  |  |  |  |
| BGC0000310 | 56.9 | 1.03E-16 | bacitracin | NRPS | bacC | antibiotic |
| BGC0002362 | 53.3 | 8.49E-17 | loseolamycin | PKS | QZS07513.1^4^ | antibiotic, herbicide |
| BGC0000319 | 47.4 | 4.90E-17 | cephamycin C | NRPS | SSCG_00146 | antibiotic |
|  | 47.4 | 4.90E-17 |  |  |  |  |
| BGC0002026 | 38.8 | 6.72E-13 | thaxteramide A1 | NRPS  PKS | thxA2 | antibiotic |
|  | 38.8 | 6.72E-13 |  |  | thxA2 |  |

^1^BGC0000770, BGC0000775 and BGC0001651 were removed as false positives, all matches were to epimerization domains within the BGC; ^2^BGC0000084 was removed as the sequenced matched outside the BGC; ^3^Sequences match to single annotated adenylation domain; ^4^Sequence matches to hypothetical protein with adenylation-like domain.

**Figure S8 –** Partial 18S rRNA sequence alignment between (**A**) *Neverita delessertiana* and 2023 egg collar sequences and (**B**) *N. duplicata* and 2023 collected egg collar sequences.
